# Supplementary material for: Crop modeling suggests limited transpiration would increase yield of sorghum across drought-prone regions of the United States
Source: Front Plant Sci. 2024 Jan 29;14:1283339. doi: 10.3389/fpls.2023.1283339 (PMC10859530; doi:10.3389/fpls.2023.1283339)
Supplement: Supplementary file 1 [file DataSheet_1.docx]

**Supplementary information**

**Table S1. Field experiments for APSIM-sorghum testing.**

| **Location** | **Year** | **Planting date** | **Target population** | **Purpose** |
| --- | --- | --- | --- | --- |
| Colby | 1997 | 05-Jun | 25000 | model evaluation |
|  | 1998 | 02-Jun | 25000 | model evaluation |
|  | 1999 | 26-May | 25000 | model evaluation |
|  | 2000 | 01-Jun | 25000 | model evaluation |
|  | 2001 | 25-May | 25000 | model evaluation |
|  | 2002 | 30-May | 25000 | model evaluation |
|  | 2003 | 29-May | 25000 | model evaluation |
|  | 2004 | 27-May | 25000 | model evaluation |
|  |  |  |  |  |
| Garden City | 1997 | 06-Jun | 35000 | model evaluation |
|  | 1998 | 22-May | 35000 | model evaluation |
|  | 1999 | 24-May | 35000 | model evaluation |
|  | 2003 | 29-May | 35000 | model evaluation |
|  | 2004 | 01-Jun | 35000 | model evaluation |
|  |  |  |  |  |
| Hays | 1997 | 23-May | 35000 | model evaluation |
|  | 1998 | 26-May | 35000 | model evaluation |
|  | 1999 | 01-Jun | 35000 | model evaluation |
|  | 2001 | 24-May | 35000 | model evaluation |
|  | 2002 | 01-Jun | 35000 | model evaluation |
|  | 2003 | 22-May | 35000 | model evaluation |
|  | 2004 | 24-May | 35000 | model evaluation |
|  |  |  |  |  |
| Manhattan | 2007 | 05-Jun | 55000 | model calibration |

**Table S2. Cultivar parameters for a default cultivar for non-LT and LT traits. Parameters tt_endjuv_to_init (*), Tpla_prod_coef (**), and Tpla_inflection (***) for a short-season sorghum are presented in the table. Parameters for medium- and full season sorghum are indicated below the table.**

| **Parameter name** | **Unit** | **Default** | **non-LT** | **LT** |
| --- | --- | --- | --- | --- |
| tt_emerg_to_endjuv | (°C day) | 100 | 100 | 100 |
| est_days_endjuv_to_init | () | 15 | 20 | 20 |
| pp_endjuv_to_init |  | 30 | 30 | 30 |
| tt_endjuv_to_init* | (°C day) | 138 | 138 | 138 |
| photoperiod_crit1 | (hours) | 12.3 | 0 | 0 |
| photoperiod_crit2 | (hours) | 14.6 | 13.5 | 13.5 |
| photoperiod_slope | (°C hour^-1^) | 25 | 0 | 0 |
| tt_flower_to_maturity | (°C day) | 695 | 810 | 810 |
| tt_flag_to_flower | (°C day) | 100 | 80 | 80 |
| tt_flower_to_start_grain | (°C day) | 30 | 50 | 50 |
| tt_maturity_to_ripe | (°C day) | 1 | 1 | 1 |
| Main_stem_coeff | (°C^-1^) | 2.95 | 2.95 | 2.95 |
| Tpla_prod_coef** | (°C^-1^) | 0.015 | 0.015 | 0.015 |
| Tpla_inflection*** | (°C) | 320 | 320 | 320 |
| Spla_prod_coef | (°C^-1^) | 0.007 | 0.005 | 0.005 |
| dm_per_seed | (g) | 0.0008 | 0.00099 | 0.00099 |
| plant canopy height | (mm) | 0 2000 | 0 1200 | 0 1200 |
| limited transpiration | (mm h^-1^) | - | - | 0.9 |

* medium-season: 190, full season: 240

** medium-season: 0.018, full season: 0.018

*** medium-season: 355.7, full season: 400.8

**Table S3. Analysis of variance for the main effects trait (G_T_), maturity group** (**G_M_), environment** (**E_W_), and planting date (M_P_) and its interaction on simulated variables.**

| **Sour. of variation** | **df** | ***p*-val^1^** | ***p*-val^2^** | ***p*-val^3^** | ***p*-val^4^** | ***p*-val^5^** | ***p*-val^6^** |
| --- | --- | --- | --- | --- | --- | --- | --- |
| Trait (G_T_) | 1 | 2×10^-16^ | 0.8 | 2×10^-16^ | 2×10^-16^ | 2×10^-16^ | 2×10^-16^ |
| Maturity (G_M_) | 2 | 2×10^-16^ | 2×10^-16^ | 2×10^-16^ | 2×10^-16^ | 2×10^-16^ | 2×10^-16^ |
| Planting date (M_P_) | 2 | 2×10^-16^ | 2×10^-16^ | 2×10^-16^ | 2×10^-16^ | 2×10^-16^ | 2×10^-16^ |
| Environment (E_W_) | 3 | 2×10^-16^ | 2×10^-16^ | 2×10^-16^ | 2×10^-16^ | 2×10^-16^ | 1×10^-5^ |
| G_T_ : G_M_ | 2 | - | - | 0.09 | - | 4×10^-7^ | - |
| G_T_ : M_P_ | 2 | - | - | - | - | - | - |
| G_T_ : E_W_ | 3 | 4×10^-7^ | 2×10^-16^ | 2×10^-16^ | 2×10^-16^ | 2×10^-16^ | 9×10^-5^ |
| G_M_ : M_P_ | 4 | 2×10^-16^ | 0.07 | - | 0.4 | 0.03 | 1×10^-9^ |
| E_W_ : M_P_ | 6 | 2×10^-16^ | 2×10^-16^ | 2×10^-16^ | 2×10^-16^ | 2×10^-16^ | 4×10^-5^ |
| E_W_ : G_M_ | 6 | 2×10^-16^ | 0.01 | 2×10^-16^ | 2×10^-16^ | 2×10^-16^ | 2×10^-16^ |
| G_T_ : G_M_ : M_P_ | 6 | - | - | - | - | - | - |
| G_T_ : E : M_P_ | 6 | - | - | - | - | - | - |
| G_T_ : G_M_ : E_W_ | 6 | - | - | 0.002 | - | - | - |
| G_M_ : E_W_ : M_P_ | 6 | 0.01 | 0.04 | - | 1×10^-4^ | 0.02 | - |

^1^ Significance for grain yield, ^2^ total transpiration, ^3^ transpiration at flowering time, ^4^ soil water content at flowering, ^5^ biomass at flowering time, and ^6^ water productivity.

- not applicable; * (*𝛼* = 0.05), **(*𝛼* = 0.01), ***(*𝛼* = 0.001)

| **Trait (G_T_)** | **Envirom**  **(E)** | **Grain yield**  **(Mg ha^-1^)** | **Group** |
| --- | --- | --- | --- |
| non-LT | ED | 3.9 ± 0.07 | a |
| LT | ED | 4.3 ± 0.07 | b |
| non-LT | MD | 4.1 ± 0.07 | a |
| LT | MD | 4.5± 0.07 | b |
| non-LT | LD | 4.2 ± 0.08 | a |
| LT | LD | 4.6 ± 0.08 | b |
| non_LT | WW | 4.8 ± 0.08 | a |
| LT | WW | 5.0 ± 0.08 | b |

**Table S4. Stepwise regression for simulated grain yield considering the main effects:trait (G_T_), maturity group** (**G_M_), environment** (**E_W_), and planting date (M_P_) with four-way interaction.**

G_T_ : trait

G_M_: maturity

E_W_: cluster_41

M_P_: pd

> step.model

Backward reduced random-effect table:

Eliminated npar logLik AIC LRT Df Pr(>Chisq)

<none> 74 -96385 192919

(1 | year:loc) 0 73 -105116 210378 17462 1 < 2.2e-16 ***

---

Signif. codes: 0 ‘***’ 0.001 ‘**’ 0.01 ‘*’ 0.05 ‘.’ 0.1 ‘ ’ 1

Backward reduced fixed-effect table:

Degrees of freedom method: Satterthwaite

Eliminated Sum Sq Mean Sq NumDF DenDF F value Pr(>F)

trait:maturity:pd:cluster_41 1 275451 22954 12 11395 0.0645 1.00000

trait:pd:cluster_41 2 129350 21558 6 11407 0.0606 0.99912

trait:maturity:pd 3 167060 41765 4 11413 0.1175 0.97636

trait:maturity:cluster_41 4 448648 74775 6 11417 0.2104 0.97369

trait:maturity 5 777644 388822 2 11423 1.0945 0.33475

trait:pd 6 928393 464197 2 11425 1.3066 0.27078

trait:cluster_41 0 11513942 3837981 3 11427 10.8024 4.388e-07 ***

maturity:pd:cluster_41 0 8954788 746232 12 11430 2.1004 0.01396 *

---

Signif. codes: 0 ‘***’ 0.001 ‘**’ 0.01 ‘*’ 0.05 ‘.’ 0.1 ‘ ’ 1

Model found:

yield ~ trait + maturity + pd + cluster_41 + (1 | year:loc) + maturity:pd + trait:cluster_41 + maturity:cluster_41 + pd:cluster_41 + maturity:pd:cluster_41

| **A** | 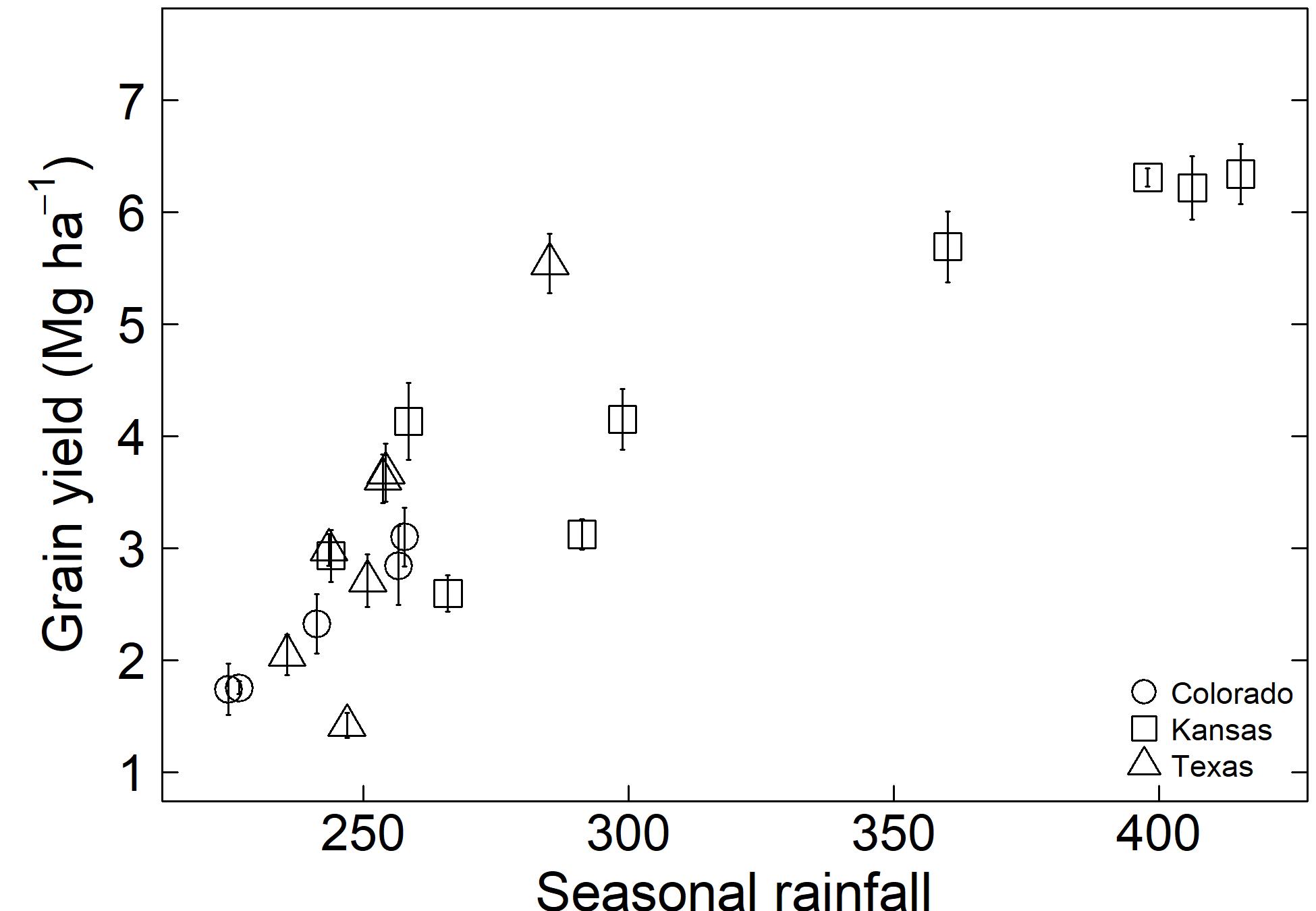 |
| --- | --- |
| **B** | 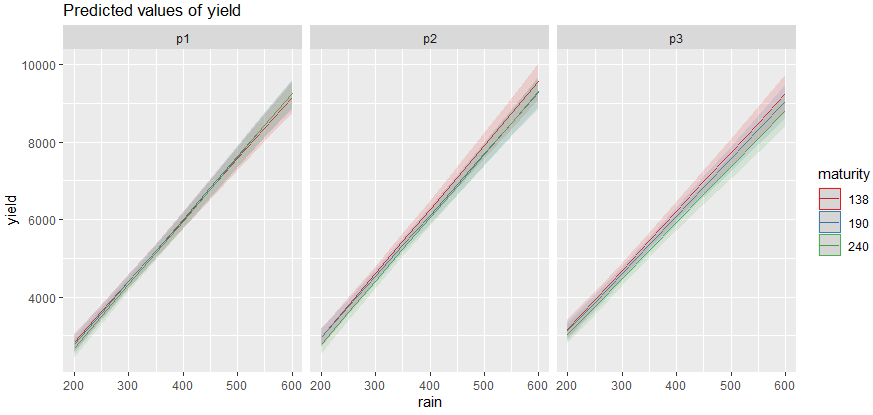 |

**Figure S1. The effect of seasonal precipitation on grain yield for a sorghum with non-LT trait.** (A) Grain yield for a simulated sorghum with non-LT trait. Simulations were conducted for three maturity groups on three planting dates. Each point represents the mean of 33 years and vertical lines indicate interannual variability (standard deviation). (B) The interaction of maturity group and planting date across precipitation gradients for a sorghum with non-LT trait.


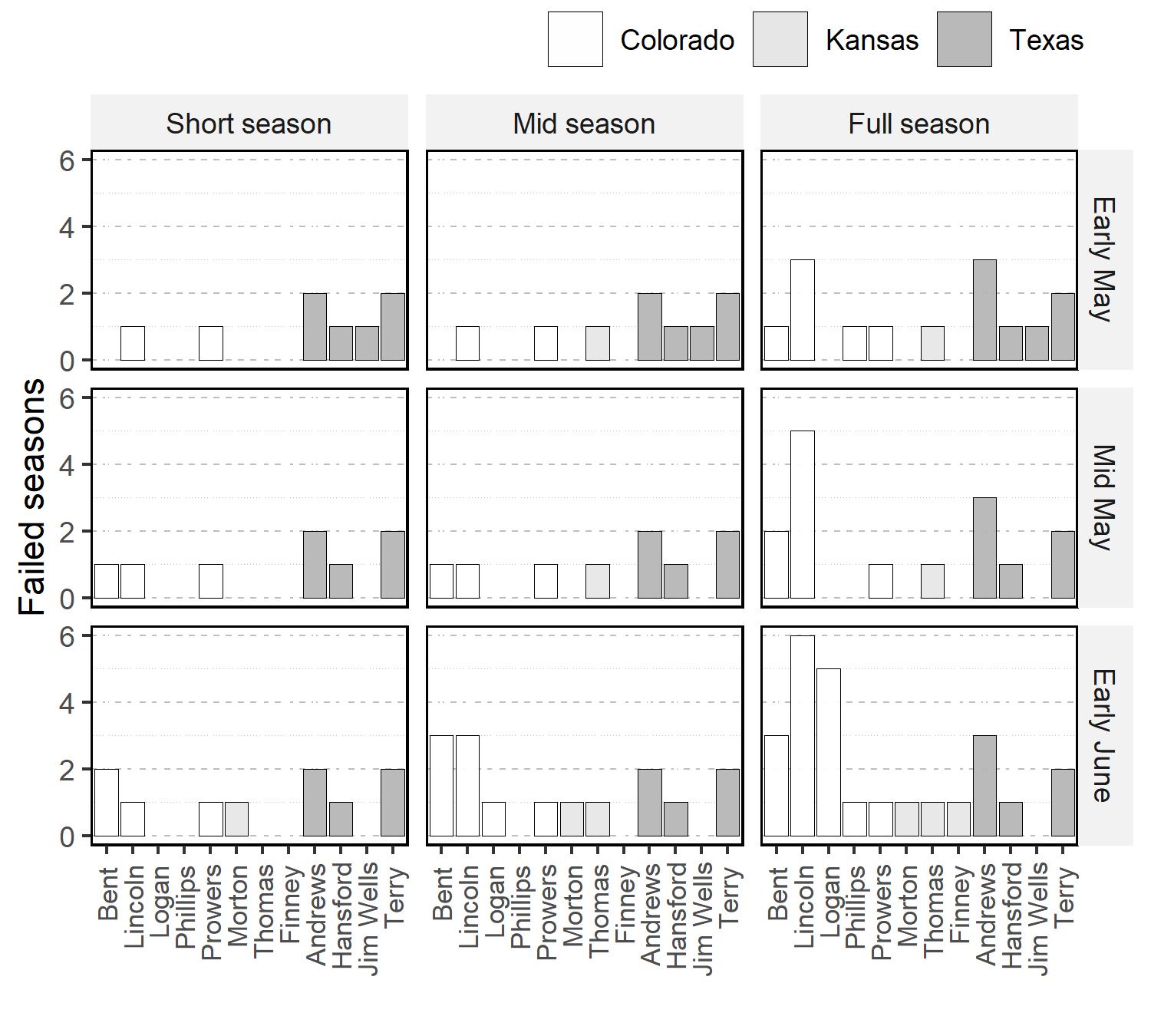


**Figure S2. Number of failed simulated seasons in the study site. A failed season indicated that the crop did not reach the vegetative or reproductive stage.**

| **A**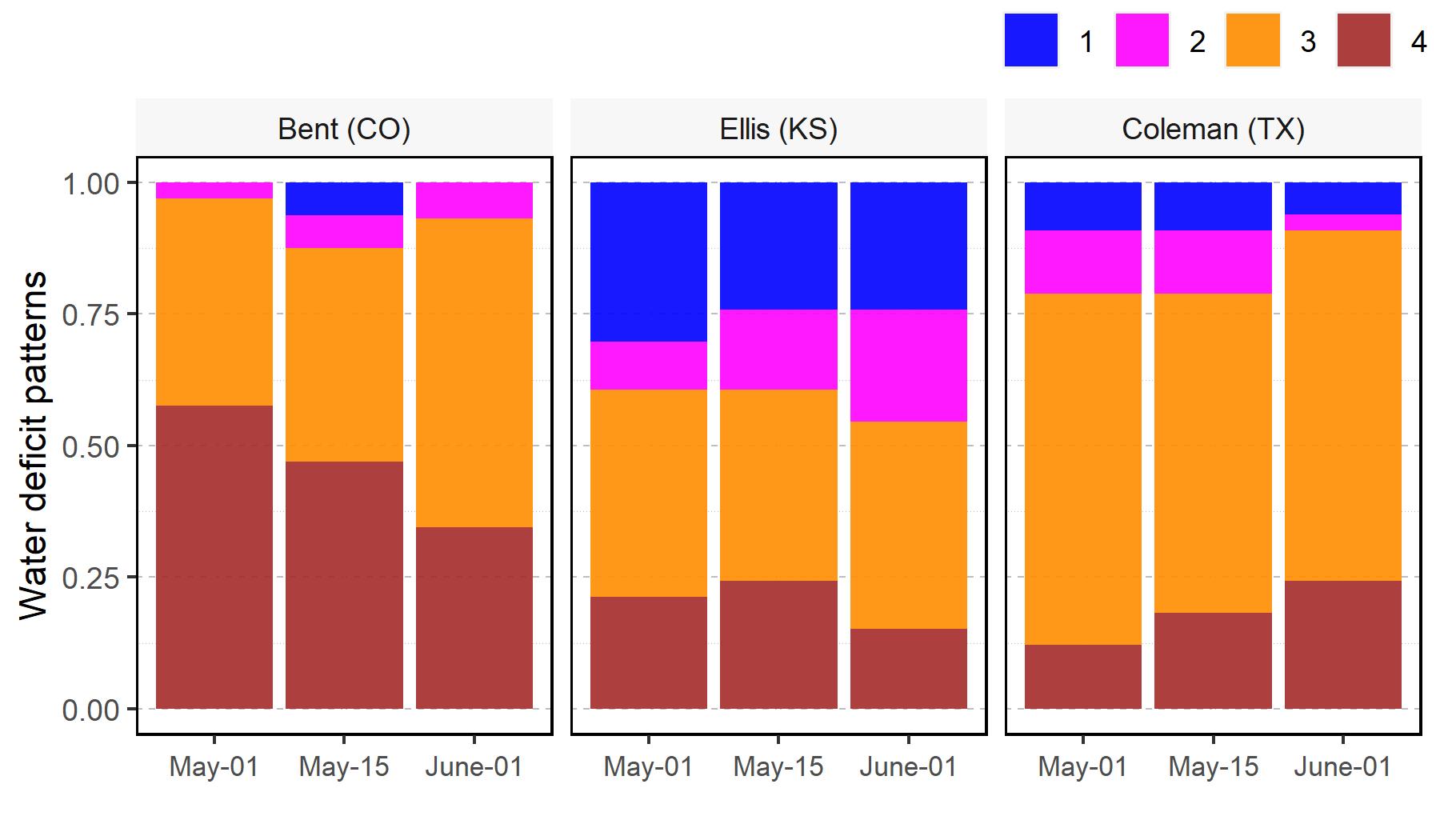 | **B**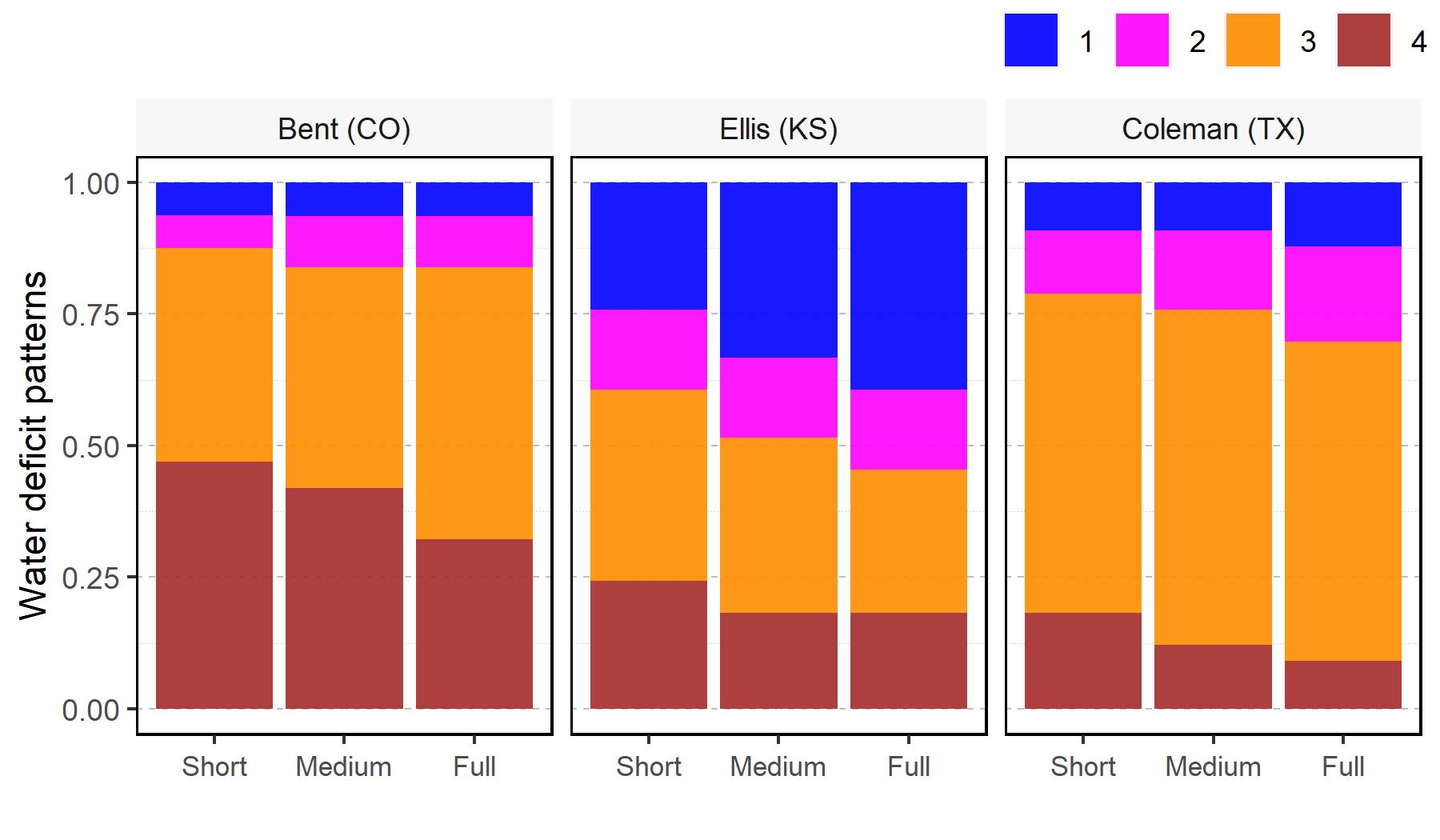 |
| --- | --- |
| **C**  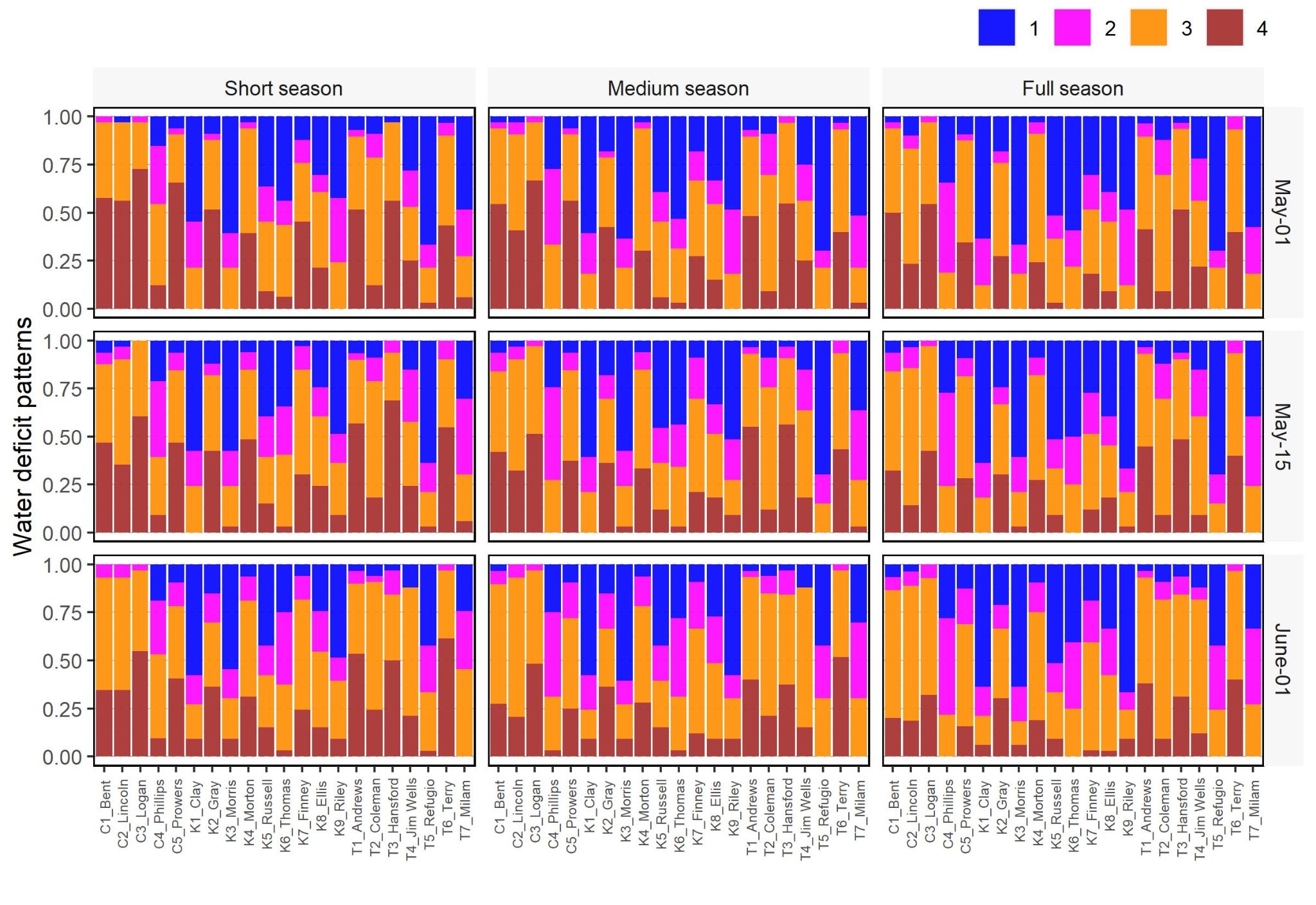 | |

**Figure S3. Frequency of water deficit scenarios in the study site.** (A) The effect of planting date on water deficit patterns for a medium season variety in Bent (CO), Ellis (KS), and Colleman (TX). (B) The effect of maturity groups on water deficit patterns for a planting date in mid-May in Bent (CO), Ellis (KS), and Colleman (TX). (C) Frequency of water deficit patterns for different maturity groups and planting dates.
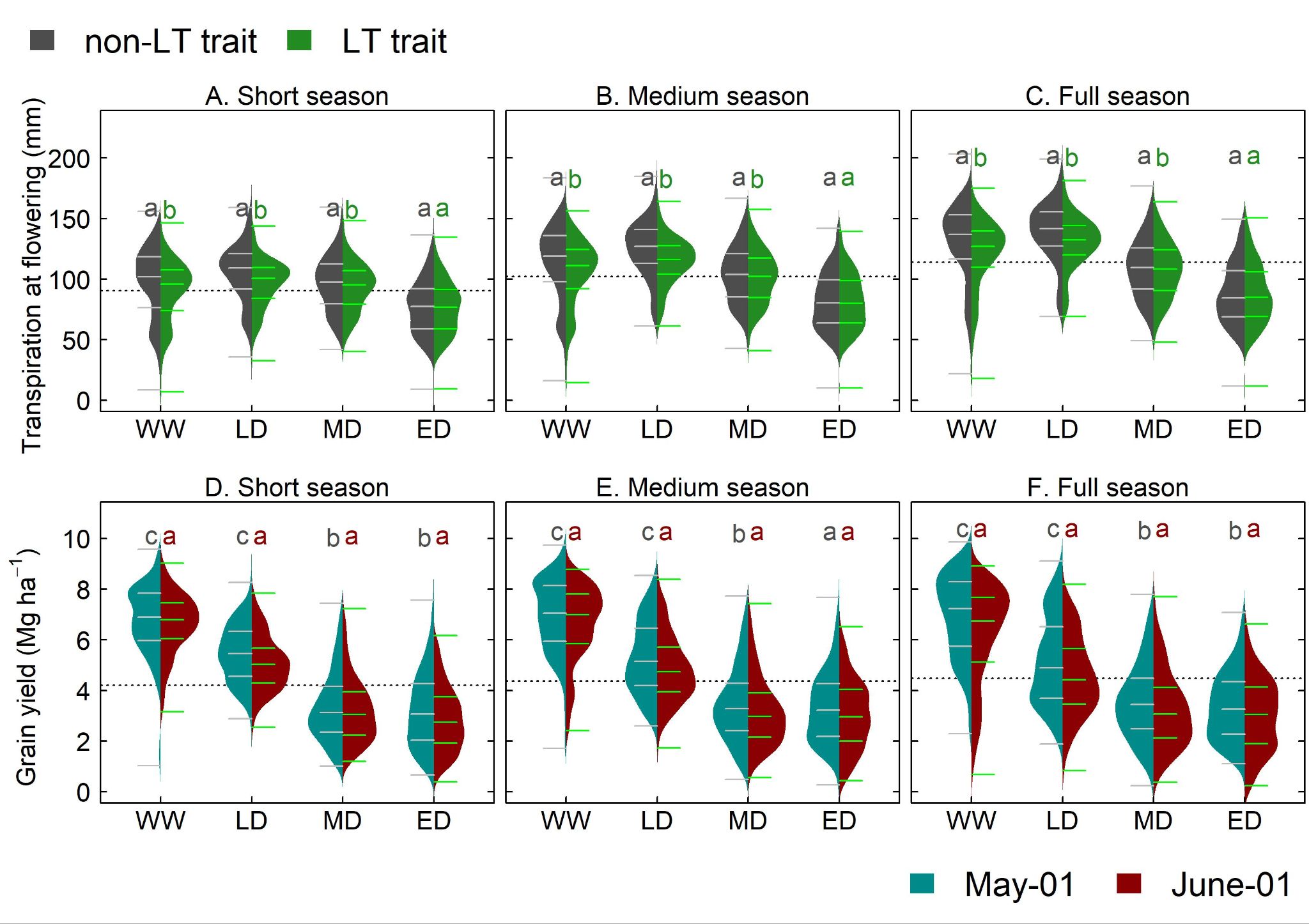


**Figure S4. Water budgets for simulated sorghum with non-LT and LT traits.** (A) transpiration at flowering time for a short-, (B) medium- and (C) full-season sorghum across the study system. (D) Grain yield for a short-, (E) medium and (F) full-season sorghum across the study system. WW: Well-watered or light stress at grain filling; LD: late drought; MD: mid-season drought; and ED: early drought (Figure 5A).


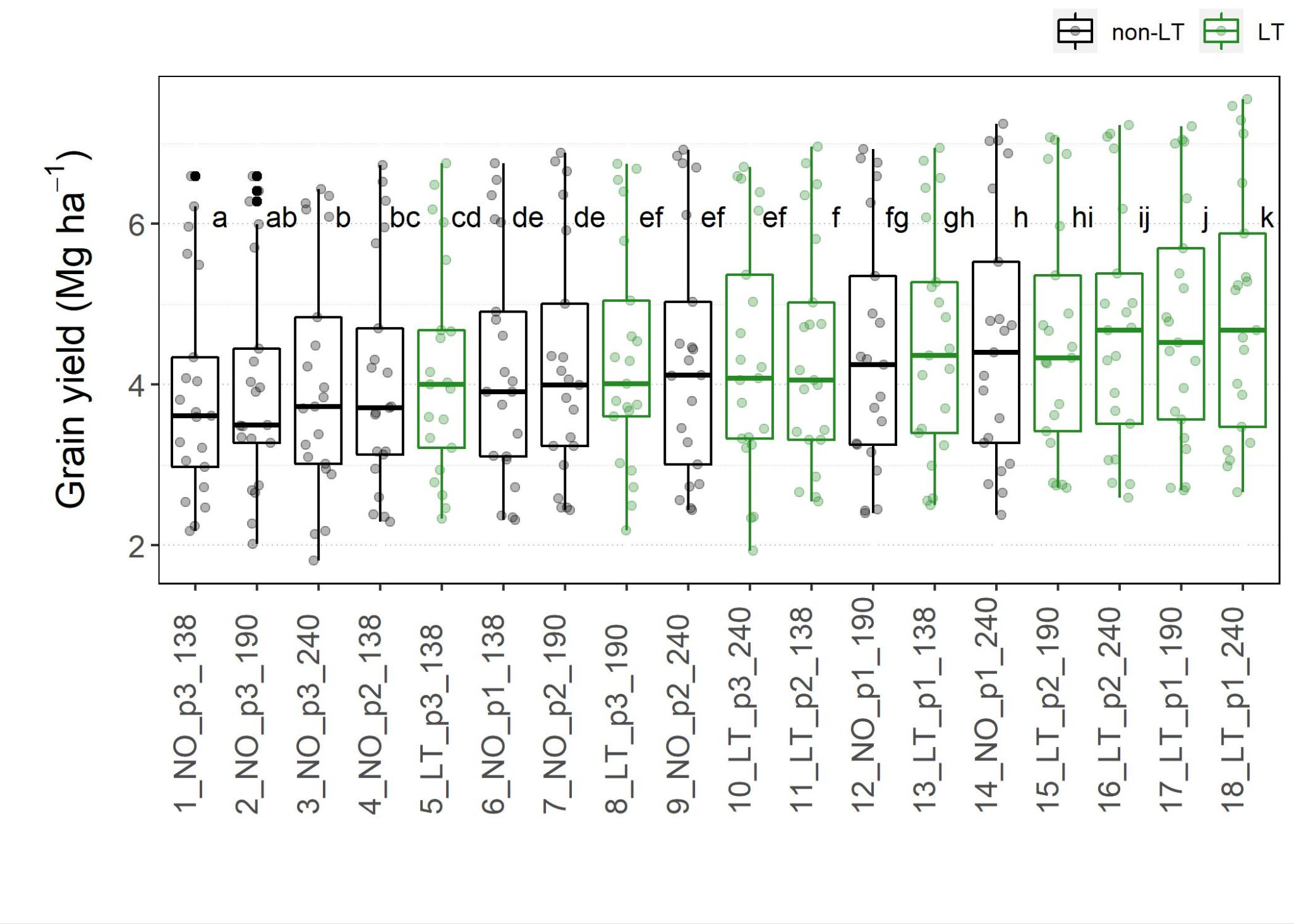


**Figure S5. Grain yield for maturity group (G_M_)** × **planting date (M_P_)**. G_M_ is represented via 138, 190 and 240 for early, medium-, and full-season sorghum, respectively. M_P_ is represented via p1, p2, and p3 for May 01, May 15 and June 01, respectively,

**
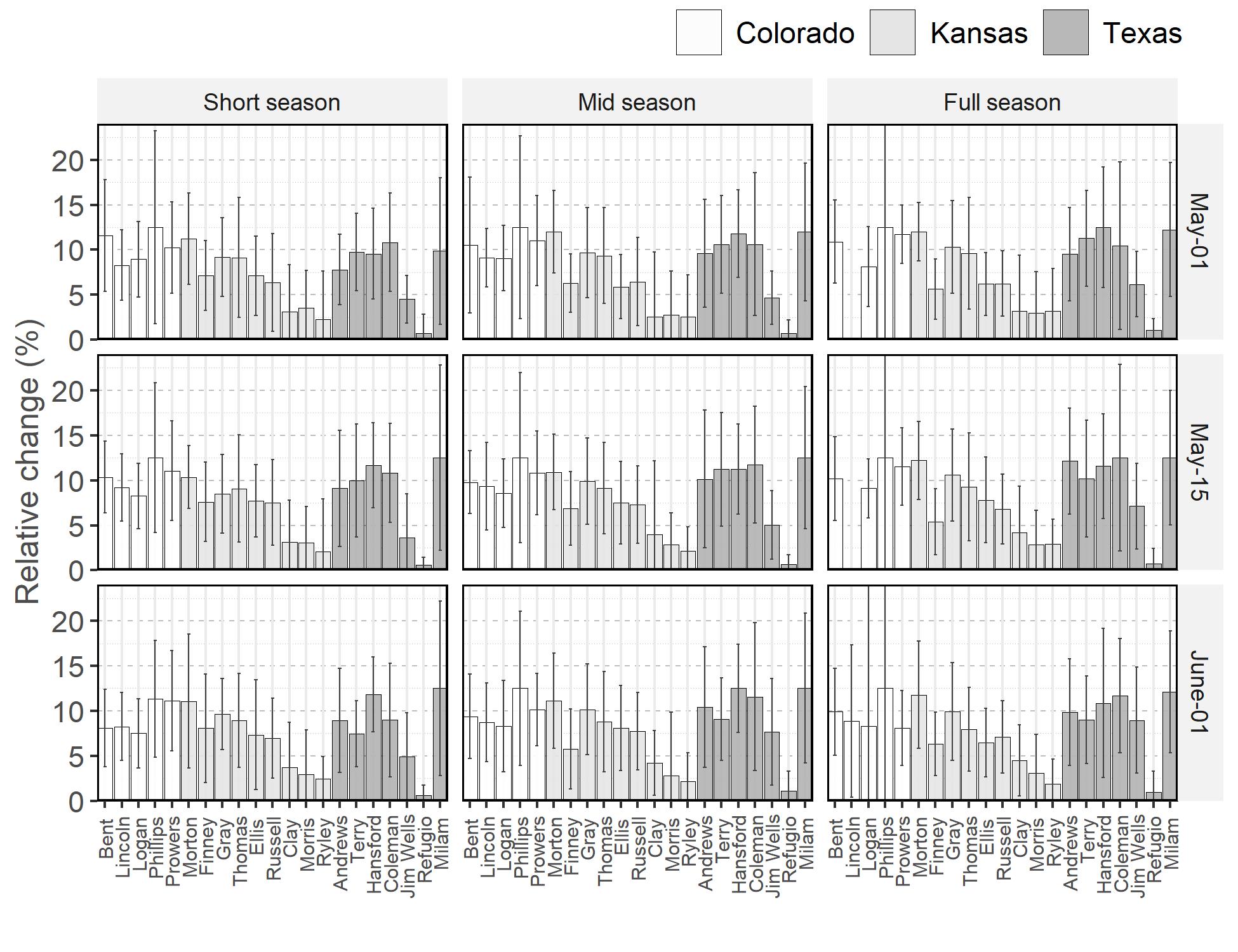
**

**Figure S6. Simulated mean relative change in grain yield for sorghum with LT trait.** Each barplot represents the mean from 1986 to 2018 and vertical lines indicate interannual variability (standard deviation).
